# Platelet Indices and Hypertension: Results from Shahedieh Cohort Study, Yazd, Iran

**DOI:** 10.1155/2024/3705771

**Published:** 2024-02-13

**Authors:** Fateme Shakeri Shamsi, Moslem Taheri Soodejani

**Affiliations:** Center for Healthcare Data Modeling, Departments of Biostatistics and Epidemiology, School of Public Health, Shahid Sadoughi University of Medical Sciences, Yazd, Iran

## Abstract

**Introduction:**

Hypertension is one of the most important diseases worldwide. In this study, we aim to demonstrate the relationship between platelet indices and hypertension.

**Materials and Methods:**

We studied 9448 people in the age range of 30 to 70 years. We assessed their hypertension status, platelet count (PLT), mean platelet volume (MPV), platelet distribution width (PDW), smoking, cardiovascular disease history, diabetes status, body mass index, and creatinine levels. Hypertension status was assessed qualitatively. All platelet indices were categorized by quartiles. We then used logistic regression to predict the relationship between these indices and hypertension.

**Results:**

PDW index and hypertension had a statistically significant relationship in the second quartile (16.2 fL < PDW ≤ 16.7 fL) in 30 to 40 years old (AOR: 0.225, 95% CI: 0.063–0.806), in the fourth quartile in 50 to 60 years old (AOR = 1.532, 95% CI: 1.048–2.238), and in all the quartiles of the age range of over 60 years. PLT index had a positive relationship (AOR = 3.147, 0.95% CI: 1.163–8.516) in 30 to 40 years old in the fourth quartile vs. the first quartile. A positive relationship was obtained in the third and fourth quartiles of PLT and the age range of 40 to 50 years, respectively (AOR = 2.063, 0.95% CI: 1.162–3.662) and (AOR = 2.204, 0.95% CI: 1.220–3.981).

**Conclusion:**

According to the results of this study, some platelet indices could be correlated with hypertension, so we may be able to reduce the burden of this disease.

## 1. Introduction

According to the World Health Organization (WHO), more than one billion people (25% of men and 20% of women) worldwide have hypertension. This disease also causes about half of strokes and ischemic heart diseases and more than 7 million deaths worldwide [1]. On average, the prevalence of hypertension in the Eastern Mediterranean region is about 21%, which is expected to increase to 30% in 2025. In Iran, due to reasons such as changing eating habits, an aging population, increasing urbanization, and obesity, the prevalence of hypertension is relatively high. Despite the improvement in diagnosis and control, about 23% of people with hypertension and more than a quarter of Iranian adults have prehypertension [2–4].

Most cases of hypertension do not have significant symptoms until severe medical illnesses such as stroke, heart attack, or chronic kidney disease occur. For this reason, prevention, early detection, and control of hypertension are of great importance [1]. Hypertension is one of the potential risk factors for cardiovascular diseases. One factor stated in various studies is the relationship between cardiovascular diseases and platelets [5–7], and this has led to the hypothesis of the relationship between platelets and hypertension.

Platelets are small, nonnucleated cells, the number of which is indicated by the platelet count (PLT). Platelets can cause thrombotic disorders through accumulation and biological adhesion processes [8]. The aggregation and reactivity of larger platelets are greater than smaller platelets, and the size of these platelets is measured by the mean platelet volume (MPV) index [9]. MPV is a determining factor and has a positive relationship with arterial stiffness [10] and is also a powerful predictor of cardiovascular mortality in people with acute coronary syndrome [11]. Another platelet index is the platelet distribution width (PDW), whose high value indicates the nonuniformity of the platelet volume [12]. PDW and MPV are indicators of platelet activation (PDW more specifically) [13], and platelet activation is one of the factors causing thrombotic events, one of the main risks of cardiovascular disease [14, 15].

Despite the high prevalence of hypertension and the asymptomatic nature of this disease, early diagnosis reduces the burden of this disease. Considering the relationship between platelets and cardiovascular diseases, one of the factors that can be related to hypertension is platelet indices. To investigate this hypothesis, previous studies focused on one or two platelet indices and reported controversial results. Also, limited studies have been carried out in this field in Iran. Therefore, this study aims to investigate the relationship between platelet indices, if any, with hypertension based on age subgroups in the central region of Iran.

## 2. Material and Methods

### 2.1. Data Accessibility

This study was conducted cross-sectionally using the data of a prospective cohort in the Shahedieh region of Yazd, Iran, in 2015. The Shahedieh cohort was part of a PERSIAN Prospective Cohort Study that was conducted to investigate noncommunicable diseases in Iran. The complete information has already been described elsewhere [16]. Sampling was carried out by multistage cluster random method. Participants entered the study with informed consent, and for illiterate participants, consent was obtained from their legal representatives. Blood samples were collected from eligible participants in a fasting state. Demographic characteristics and lifestyle information were evaluated using a valid questionnaire. Anthropometric indices and blood pressure were measured and recorded by trained interviewers after resting subjects. The characteristics of the participants including age, sex, anthropometric indicators, smoking, and self-reported history of diabetes and cardiac diseases were extracted from the cohort study.

### 2.2. Study Design

The data of 9956 people were extracted using questionnaires, clinical examinations, laboratory, and paraclinical tests. Then, the missing data for blood pressure variables and platelet indices were excluded from the study, and finally, 9448 people aged 30 to 70 years old were included in the study. According to the guidelines of the WHO, hypertension is defined as a systolic blood pressure of 140 mm Hg or higher and a diastolic blood pressure of 90 mm·Hg or higher [17]. People who had both systolic and diastolic blood pressure above normal were considered as hypertensive patients, and others were considered healthy. Using the collected samples, the results of creatinine, triglyceride, cholesterol, PDW, MPV, and PLT were extracted. Three available platelet indices (PDW, MPV, and PLT) were divided into quartiles to enter the analysis. A cholesterol level above 200 mg/dL was defined as high cholesterol. Creatinine level from 0.8 to 1.39 mg/dL was considered as normal and with two upper and lower limits. A triglyceride level of 50–150 mg/dL was classified as normal. Self-reported diabetes, use of hypoglycemic drugs, or having FBS ≥126 mg/dL was defined as diabetes. The urine glucose level was analyzed as positive or negative. Normal weight was defined as BMI between 18.5 and 25 kg/m^2^, overweight between 25 and 30 kg/m^2^, and more than 30 kg/m^2^ as obese [18]. To evaluate the smoking status according to the questionnaire, the participants were asked: Have you smoked at least 100 cigarettes in your entire life? People who answered “yes” were classified as smokers and those who answered “no” were classified as nonsmokers.

### 2.3. Data Analysis

Quantitative variables were qualitatively classified, numbers and percentages were used to describe qualitative variables, and a chi-square test was used to analyze them. For regression analysis, people were classified into 4 age levels. Univariate regression analysis was performed to evaluate the crude odds ratio between blood pressure and platelet indices (in quartiles) as well as other risk factors. Potential confounding factors such as sex, smoking, BMI, cardiac disease, diabetes, creatinine levels, urine glucose, triglycerides, and cholesterol were entered into the multivariate regression model. The odds ratio of blood pressure with quartile platelet indices was obtained as adjusted. Statistical analyses were performed using STATA software (version 17). A statistical significance level of 0.05 was considered.

## 3. Results

After cleaning missing data, 9448 participants (4721 women and 4727 men) were included in the study. In all age groups, men were more prone to hypertension than women. Among the study participants, 18% were between 20 and 30 years old, 35% were 30 to 40 years old, 28% were 40 to 50 years old, and 18% were over 60 years old. The proportion of people with hypertension increased with each decade of age. Among the people with hypertension, 0.42% were thin (BMI <18.5 kg/m^2^), 11.01% had a normal BMI (18.5–25 kg/m^2^), 38.48% were overweight (BMI: 25–30 kg/m^2^), and 50.07% were obese (BMI >30 kg/m^2^). This shows that a higher proportion of obese and overweight people have hypertension. 8.57% of smokers and 7.05% of nonsmokers had hypertension. Among the people with hypertension, 12.16% had cardiac disease and 31.18% had diabetes. 8.59% of people with high blood cholesterol and 6.74% of people with normal blood cholesterol had hypertension (Table 1).

The data of MPV, PDW, and PLT values were classified into quartiles, and the first quartile was considered as a reference. We performed univariate logistic regression analysis for identified risk factors. PDW index in the second quartile, in the two age ranges of 30–40 years (OR = 0.205, CI: 0.059–0.715) and over 60 years (OR = 0.660, CI: 0.452–0.963), has a negative and significant relationship with blood pressure. In the third quartile of PDW, there was a negative and significant relationship only in the age range of over 60 years (OR = 0.660, CI: 0.460–0.946). The fourth quartile had different and significant results with blood pressure in two age ranges of 50 to 60 years (OR = 1.679, CI: 1.164–2.421) and over 60 years (OR = 0.685, CI: 0.483–0.972). MPV index had no significant relationship in any of the age ranges. The PLT index showed a significant and positive relationship with blood pressure only in the age range of 40 to 50 years in the third (OR = 1.950, CI: 1.132–3.358) and fourth (OR = 2.021, CI: 1.158–3.447) quartiles. High triglyceride levels (triglyceride >151 mg/dL) in age groups below 60 years had a positive and significant relationship with blood pressure. High creatinine (creatinine >1.40 mg/dL) had a significant relationship with blood pressure only in the age group of 50 to 60 years (OR = 1.568, CI: 1.149–2.140). Cholesterol in the age range of 30 to 50 years had a positive and significant relationship with blood pressure. BMI between 18.5 and 25 was considered as a reference, and people who were overweight (BMI = 25–30 kg/m^2^) had a positive and significant relationship with blood pressure in the age ranges of 40 to 60 years. Obese people (BMI >30 kg/m^2^) had a positive and significant relationship in all age ranges. Smoking in the age range of 50 to 60 years showed a positive and significant relationship with blood pressure (OR = 1.424, CI: 1.083–1.871). Diabetes showed a positive and significant relationship with blood pressure in the age ranges of 40 to 50 years (OR = 2.743, CI: 1.808–4.162) and also 50 to 60 years (OR = 1.451, CI: 1.101–1.913) (Table 2).

After adjusting for confounding factors, blood pressure had a negative relationship (OR = 0.225, CI: 0.063–0.806) with the PDW index in the second quartile and the age range of 30 to 40 years. In the fourth quartile of PDW and the age range of 50 to 60 years, a positive and significant relationship was obtained (OR = 1.532, CI: 1.048–2.238), and in the age range of over 60 years in all PDW quartiles, the relationship was significant and negative. Even after adjusting for confounding factors, the MPV index did not show a significant relationship with blood pressure. PLT index in the fourth quartile and age range of 30 to 40 years had a positive and significant relationship (OR = 3.147, CI: 1.163–8.516). A positive relationship was obtained in the third and fourth quartiles of PLT and the age range of 40 to 50 years, respectively (OR = 2.063, CI: 1.162–3.662) and (OR = 2.204, CI: 1.220–3.961) (Table 3).

## 4. Discussion

In our study, the platelet activation parameter, PDW, showed a positive relationship with hypertension in the age range of 50 to 60 years and the last quartile, but a protective relationship with hypertension in the age range of over 60 years and the second quartile in the age range of 30 to 40 years was obtained. Regarding the relationship between hypertension and PDW, there are few studies with controversial results. In a study of 73,000 participants, with the increase of PDW quartiles in women, the incidence of isolated systolic blood pressure increased, but in men, the difference between PDW quartiles was insignificant [19]. A longitudinal study using the quadratic inference function method found a negative relationship between systolic blood pressure and PDW, but no relationship was found with diastolic blood pressure except in the second quartile in men [20]. A study with participants of European descent and using the multivariate Mendelian randomization method reported a causal relationship between PDW and diastolic and systolic blood pressures, indicating a positive relationship between activated platelets and hypertension [21]. In our study, people in different age ranges were investigated, and the use of different methods in these studies could be one of the reasons for the results not being the same.

The presence of larger and more reactive platelets increases MPV [22]. According to the results of our study, MPV was not significantly related to hypertension in any of the quartiles. The results of some previous studies were in same as our results, but some others have reported different results. In a study with about 44,000 participants, no relationship between MPV subgroups and hypertension was shown after adjusting for blood-related background variables [23]. Also, the results of a study comparing two healthy control groups and people with hypertension showed that in the group of people with hypertension, the mean number of MPV increased significantly, but no significant correlation was found between MPV and blood pressure indicators [24]. However, the results of a cohort study in China showed that in the highest quartile of MPV compared to the lowest quartile, the average values of systolic and diastolic blood pressure of people are higher, and people who had higher MPV at the beginning of the study were diagnosed with hypertension [25]. In a case-control study, higher MPV values were obtained in patients with hypertension and prehypertension compared to control subjects [26]. The potential mechanism of MPV changes in people with hypertension is not clear, but one of the reasons for the inconsistency of the study results is the existence of confounders that have not been controlled in the same way in different studies, and conducting these studies on different ethnic groups can be another reason for this difference results.

The changes in PLT and blood pressure in our study were found to be significant only in the age range of 30 to 50 years. In these age ranges, the third and fourth quartiles of PLT had a significant and positive relationship with hypertension, which findings are consistent with the results of previous studies. With the increase in blood pressure, the endothelium is damaged, and as a result of this damage, PLT starts to be produced and activated, and this increases PLT in the place of the damaged blood vessel [24]. The results of a longitudinal study in China showed a positive relationship between PLT and diastolic blood pressure [20]. Also, a study estimated the relationship between PLT and hypertension with the Mendelian randomization method, in which univariately, PLT levels were associated with increased blood pressure, and in the multivariate model, after collinearity correction, the effect of PLT on hypertension remained strong [21]. However, contrary to these results, another study compared two hypertensive groups and healthy controls in terms of PLT, PLT was not significantly different in the two groups, and the duration of hypertension showed a negative correlation with PLT [24].

Platelets of hypertensive patients have different lipid contents and fluidity than those with normal blood pressure, and this difference has been confirmed by liquid chromatography-mass spectrometry [27]. Platelets express many receptors that indicate the role of platelets in thrombosis, such as granule release and deformation. Larger platelets have more thrombotic potential than smaller platelets and contain denser granules and more active enzymes, thus accelerating thrombus formation and leading to clinical events such as acute coronary syndrome [28, 29]. Platelet indices are diagnostic biomarkers and are part of basic and inexpensive blood tests that can be useful indicators for the early detection of thromboembolic disease. In order to use these indicators as one of the prognostic methods of hypertension, it is necessary to conduct large and cohort studies to achieve clear results in this regard.

This study measured the relationship between platelet indices and hypertension in Iranian adults with a large sample size and using standard tools and methods. Among the other strengths of this study, we can mention the proportional ethnic and age distribution of the society.

Different conditions affect platelet indices, and in the current study, people with secondary hypertension and taking platelet inhibitor drugs were not considered, which is one of the limitations of our study. Also, some lifestyle factors such as physical activity and salt intake were not adjusted for the participants. Among the possible reasons for the lack of correlation between hypertension and some platelet indices in this study, it can be mentioned that in our study, people with high systolic and diastolic blood pressure were included in the analysis as hypertension and were not separately analyzed.

## 5. Conclusion

PDW and PLT could be correlated with hypertension, but the result of examining the relationship between MPV and hypertension in this study was not significant. However, prospective studies are needed to achieve exact results in using platelet indices to predict hypertension.

## Figures and Tables

**Table 1:** Relationship between hypertension and some major risk factors.

Variable		Hypertension		*P* value^∗^
		No	Yes	
Age groups (year)	30–39.9	1662 (97.82%)	37 (2.18%)	<0.01
	40–49.9	3204 (95.87%)	138 (4.13%)	
	50–59.9	2391 (90.16%)	261 (9.84%)	
	60≤	1492 (85.01%)	263 (14.99%)	

Sex (*n* (%))	Female	4406 (93.32%)	315 (6.68%)	<0.01
	Male	4343 (91.88%)	384 (8.12%)	

Smoke cigarette (*n* (%))	No	6796 (92.95%)	515 (7.05%)	0.02
	Yes	1952 (91.43%)	183 (8.57%)	

BMI (kg/m^2^)	≤18.49	111 (97.37%)	3 (2.63%)	<0.01
	18.5/24.9	2063 (96.40%)	77 (3.60%)	
	25/29.9	3731 (93.27%)	269 (6.73%)	
	30≤	2838 (89.02%)	350 (10.98%)	

Cardiac disease (*n* (%))	No	8074 (92.93%)	614 (7.07%)	<0.01
	Yes	675 (88.82%)	85 (11.18%)	

Diabetes (*n* (%))	No	7265 (93.79%)	481 (6.12%)	<0.01
	Yes	1484 (87.19%)	218 (12.81%)	

Creatinine (mg/dL)	Low min/0.79	180 (95.24%)	9 (4.76%)	<0.01
	Normal 0.80/1.39	7325 (92.93%)	557 (7.07%)	
	High 1.40/max	1236 (90.29%)	133 (9.71%)	

Glucose (*n* (%))	Negative	7982 (93.11%)	590 (6.89%)	<0.01
	Positive	568 (84.78%)	102 (15.22%)	

Triglyceride (mg/dL)	Low min/49	123 (99.20%)	1 (0.8%)	<0.01
	Normal 50/150	4675 (94.01%)	298 (5.99%)	
	High 151/max	3943 (90.79%)	400 (9.21%)	

Cholesterol (mg/dL)	Normal ≤200	5644 (93.26%)	408 (6.74%)	<0.01
	High 201≤	3097 (91.41%)	291 (8.59%)	

PDW (fl)	Quartile 1	2234 (92.08%)	192 (7.92%)	0.02
	Quartile 2	2184 (93.41%)	154 (6.59%)	
	Quartile 3	2242 (93.41%)	158 (6.59%)	
	Quartile 4	2078 (91.42%)	195 (8.58%)	

MPV (fl)	Quartile 1	3061 (92.53%)	247 (7.47%)	0.99
	Quartile 2	1389 (92.79%)	108 (7.21%)	
	Quartile 3	2228 (92.64%)	177 (7.36%)	
	Quartile 4	2061 (92.5%)	167 (7.5%)	

PLT (×10^9^/l)	Quartile 1	2238 (93.44%)	157 (6.56%)	0.09
	Quartile 2	2189 (93.03%)	164 (6.97%)	
	Quartile 3	2177 (92.20%)	184 (7.80%)	
	Quartile 4	2137 (91.68%)	194 (8.32%)	

^∗^Chi-square test values.^∗^ shows the type of statistical test and all significance values are shown in the last column.

**Table 2:** Relationship between hypertension and platelet indices (crude OR (95% CI)).

Platelet indices	Quartiles	Age groups			
		30–39.9	40–49.9	50–59.9	60≤
PDW (fl)	Quartile 1	1	1	1	1
	Quartile 2	0.205 (0.059–0.715)^*∗*^	0.687 (0.424–1.115)	1.437 (0.983–2.101)	0.660 (0.452–0.963)^*∗*^
	Quartile 3	0.440 (0.169–1.145)	0.960 (0.614–1.501)	0.971 (0.649–1.452)	0.660 (0.460–0.946)^*∗*^
	Quartile 4	1.194 (0.560–2.544)	0.762 (0.468–1.243)	1.679 (1.164–2.421)^*∗*^	0.685 (0.483–0.972)^*∗*^

MPV (fl)	Quartile 1	1	1	1	1
	Quartile 2	2.258 (0.861–5.919)	0.608 (0.351–1.052)	0.812 (0.546–1.208)	1.457 (0.980–2.166)
	Quartile 3	1.461 (0.559–3.821)	0.678 (0.438–1.050)	0.958 (0.688–1.332)	1.380 (0.976–1.951)
	Quartile 4	2.696 (0.676–4.255)	0.719 (0.455–1.136)	0.919 (0.656–1.288)	1.383 (0.974–1.963)

PLT (×10^9^/l)	Quartile 1	1	1	1	1
	Quartile 2	0.631 (0.198–2.003)	1.529 (0.846–2.707)	1.199 (0.833–1.725)	1.044 (0.719–1.517)
	Quartile 3	1.504 (0.577–3.918)	1.950 (1.132–3.358)^*∗*^	1.166 (0.808–1.684)	1.155 (0.803–1.662)
	Quartile 4	2.121 (0.847–5.312)	2.021 (1.185–3.447)^*∗*^	1.222 (0.846–1.765)	1.330 (0.925–1.912)

^*∗*^ *p* < 0.05. The value of PDW quartiles. Q1: PDW ≤ 16.2 (reference), Q2: 16.2 < PDW ≤ 16.7, Q3: 16.7 < PDW ≤ 17.2, and Q4: PDW > 17.2. The value of MPV quartiles. Q1: MPV ≤ 7.5 (reference), Q2: 7.5 < MPV ≤ 7.7, Q3: 7.7 < MPV ≤ 8.1, and Q4: MPV > 8.1. The value of PLT quartiles. Q1: PLT ≤ 205 (reference), Q2: 205 < PLT ≤ 238, Q3: 238 < PLT ≤ 276, and Q4: PLT > 276.

**Table 3:** Relationship between hypertension and platelet indices (adjusted OR (95% CI)).

Platelet indices	Quartiles	Age groups			
		30–39.9	40–49.9	50–59.9	60≤
PDW (fl)	Quartile 1	1	1	1	1
	Quartile 2	0.225 (0.063–0.806)^*∗*^	0.640 (0.388–1.056)	1.353 (0.917–1.997)	0.644 (0.437–0.950)^*∗*^
	Quartile 3	0.455 (0.169–1.229)	0.870 (0.549–1.380)	0.928 (0.614–1.402)	0.657 (0.455–0.949)^*∗*^
	Quartile 4	1.030 (0.458–2.318)	0.695 (0.147–1.158)	1.532 (1.048-2.238)^*∗*^	0.679 (0.473–0.973)^*∗*^

MPV (fl)	Quartile 1	1	1	1	1
	Quartile 2	2.535 (0.952–6.750)	0.638 (0.366–1.114)	0.797 (0.532–1.196)	1.446 (0.965–2.166)
	Quartile 3	1.345 (0.476–3.796)	0.659 (0.420–1.033)	0.968 (0.690–1.357)	1.349 (0.949–1.919)
	Quartile 4	2.183 (0.848–5.621)	0.771 (0.483–1.228)	0.928 (0.656–1.314)	1.308 (0.912–1.876)

PLT (×10^9^/l)	Quartile 1	1	1	1	1
	Quartile 2	0.608 (0.188–1.961)	1.571 (0.870–2.836)	1.212 (0.835–1.758)	1.100 (0.749–1.614)
	Quartile 3	1.321 (0.477–3.656)	2.063 (1.162-3.662)^*∗*^	1.237 (0.843–1.816)	1.252 (0.854–1.837)
	Quartile 4	3.147 (1.163–8.516)^*∗*^	2.204 (1.220–3.981)^*∗*^	1.370 (0.916–2.049)	1.476 (0.992–2.198)

^*∗*^ *p* < 0.05. The value of PDW quartiles. Q1: PDW ≤ 16.2 (reference), Q2: 16.2 < PDW ≤ 16.7, Q3: 16.7 < PDW ≤ 17.2, and Q4: PDW > 17.2. The value of MPV quartiles. Q1: MPV ≤ 7.5 (reference), Q2: 7.5 < MPV ≤ 7.7, Q3: 7.7 < MPV ≤ 8.1, and Q4: MPV > 8.1. The value of PLT quartiles. Q1: PLT ≤ 205 (reference), Q2: 205 < PLT ≤ 238, Q3: 238 < PLT ≤ 276, and Q4: PLT > 276. Adjusted by triglyceride, creatinine, glucose, cholesterol, BMI, sex, smoke cigarette, diabetes, and cardiac disease.

## Data Availability

The original data used to support the findings of this study are available from the corresponding author upon request.

## Abbreviations

WHO: World Health Organization
PDW: Platelet distribution width
MPV: Mean platelet volume
PLT: Platelet count
BMI: Body mass index
FBS: Fasting blood sugar
OR: Odds ratio
CI: Confidence interval.
